# Sublingual leukocyte activation in patients with severe sepsis or septic shock

**DOI:** 10.1186/cc14103

**Published:** 2015-03-16

**Authors:** BK Fabian-Jessing, MJ Massey, MR Filbin, PC Hou, H Kirkegaard, HE Wang, DM Yealy, JA Kellum, DC Angus, NI Shapiro

**Affiliations:** 1 Beth Israel Deaconess Medical Center, Boston, MA, USA; 2 Massachusetts General Hospital, Boston, MA, USA; 3 Brigham and Women's Hospital, Boston, MA, USA; 4 Aarhus University Hospital, Aarhus, Denmark; 5 University of Alabama at Birmingham, AL, USA; 6 University of Pittsburgh, PA, USA

## Introduction

The objective of this study was to compare the number of rolling and adhered leukocytes in patients with severe sepsis/septic shock with non-infected controls. Microcirculatory flow alterations and endothelial cell dysfunction are elements of sepsis pathophysiology. Traditionally, microcirculatory emphasis has been on red blood cell vessel perfusion. However, assessment of interactions between white blood cells and endothelial cells may be another early diagnostic modality.

## Methods

We included adult (age >18 years) ED patients presenting with severe sepsis/septic shock (sepsis with elevated lactate (>4 mmol/l)) or hypotension) from the prospective clinical ProCESS trial. We studied a subset of patients with microcirculatory videos obtained along with non-infected control patients. Using a sidestream dark-field videomicroscope, we obtained image sequences from the sublingual mucosa and used video stabilization and frame averaging techniques to visualize slowly-moving leukocytes. We quantified the number of rolling and adhered leukocytes present per 1 mm × 1 mm visual field in a standardized 3-second clip. Furthermore, we extracted the total length of vessels candidate for counting of rolling/adhered leukocytes (vessels with an adequate focus). We report sample means with standard deviation and compare them with Student's *t *test.

## Results

We included a total of 64 patients with severe sepsis/septic shock and 32 non-infected controls. The mean number of adhered leukocytes per field in the sepsis group was 2.1 (SD 2.3) compared with 0.4 (SD 0.8) in the non-infected group (*P *< 0.001). This corresponded to a mean number of adhered leukocytes per unit vessel length of 0.16/mm (SD 0.22) and 0.03/mm (SD 0.06) for sepsis and non-infected groups, respectively (*P *< 0.001). For the rolling leukocytes, we observed a mean number of 27.8 (SD 19.4) in the sepsis group and 12.0 (SD 8.7) in the non-infected group (*P *< 0.001) per field. This corresponded to a mean number of rolling leukocytes per unit vessel length of 2.00/mm (SD 1.67) and 0.75/mm (SD 0.55), respectively (*P *< 0.001).

## Conclusion

Our results show a higher number of rolling and adhered leukocytes in patients with severe sepsis/septic shock when compared with non-infected controls. This also applies when taking the total vessel length in the field of view into consideration. This may hold potential as a useful tool in sepsis assessment.

